# Determinants of associated events following AZD1222 (Covishield) vaccination in a high-risk population in Nepal

**DOI:** 10.1186/s12879-022-07406-2

**Published:** 2022-05-03

**Authors:** Kapil Madi Poudel, Neha Shah, Manab Prakash, Santosh Kumar Deo, Sunita Bhandari, Tika Ram Poudel

**Affiliations:** 1 Institute of Medicine, Tribhuvan University, Maharajgunj, Kathmandu, 44600 Nepal; 2 Dhankuta Institute of Health Science, Dhankuta, 56800 Nepal; 3 BP Koirala Institute of Health Sciences, Dharan, 56700 Nepal; 4 Central Department of Economics, Tribhuvan University, Kirtipur, 44600 Nepal; 5 Birat Medical College, Biratnagar, Nepal; 6 GoldenGate International College, Tribhuvan University, Kathmandu, 44600 Nepal

**Keywords:** Adverse event, COVID-19, Covishield, Comorbidity, Socio-demographic characteristics

## Abstract

**Background:**

Vaccination is the most effective method to prevent the spread of infectious diseases and helps reduce mortality rate and economic costs associated with the pandemic. Despite these advantages, misinformation on vaccine safety and efficacy can lead to increased hesitation towards vaccination. This study reports the incidence of adverse events following Covishield vaccination, their associated factors, medication used for their management, and attitudes about vaccine safety.

**Methods:**

A cross-sectional study was conducted from the sample of Covishield-vaccinated individuals from a secondary hospital, two primary health centres, and 36 health posts in eastern Nepal. Individuals (*n* = 602) were randomly sampled from a population (*n* = 1013) who had received the first dose of Covishield, namely frontline workers and other high-risk populations. The second-round follow-up had 516 participants. Association of incidence and severity of post-vaccination events with socio-demographic variables, comorbidity status, and medication use were estimated.

**Results:**

Among the 79.9% of participants who reported adverse events after receiving the first dose, two-thirds of complaints were mild (67.4%, 95% CI 63.2–71.6) with the most common complaint being pain at the injection site (86.5%). Paracetamol or its combination with NSAIDs were used in the majority of cases (95.2%). After the second dose, only 31.2% (95% CI 27.2–35.2) reported adverse events, the overwhelming majority of which were mild (95.7%) and required a lower frequency of medication (7.5% vs. 26.0%). Adverse event following immunization were significantly associated with being 18–30 years old (χ^2^ = 16.9, *df* = 3, *p* < 0.001) and female gender (χ^2^ = 5.2, *df* = 1, p < 0.05). Prior to the first dose, 86.0% of participants (95% CI 83.3–88.8%) perceived the vaccine to be safe, and 96.0% recommended the vaccine post-vaccination, while 96.8% were interested in receiving the second dose. AEFI severity was negatively associated with vaccine recommendation to the peers (odds-ratio 0.062, p < 0.05) following the first dose, whereas, the optimistic pre-vaccination perception was associated with positive vaccine recommendation post-vaccination (odds-ratio 28.658, p < 0.01).

**Conclusions:**

Overall, vaccination-associated events were mild and majority were managed with paracetamol or its combination. Effective counselling about adverse events before vaccination should be prioritized to reduce hesitation and fear.

**Supplementary Information:**

The online version contains supplementary material available at 10.1186/s12879-022-07406-2.

## Background

The novel coronavirus disease COVID-19 is a global health catastrophe [1], that has impacted public health, economies, and people’s social interactions and daily lives worldwide [2]. It is a respiratory illness with a high infection rate [3], the possibility of infection from asymptomatic infection [4], and the ability to cause fatal complications. These characteristics combination has resulted in public policy responses worldwide, including mass vaccination, quarantines, and the closure of public spaces [5], all aimed at disrupting the chain of viral transmission.

Vaccinating vulnerable populations is the most effective method of preventing the spread of infectious diseases and thus reducing the high mortality and economic costs associated with such pandemics [6]. The COVID-19 pandemic is the first pandemic for which vaccines have been available in less than a year after the first reported case. By March 2021, 13 vaccines had been approved for use, with more than 90 others in clinical trials [7]. In Nepal, Covishield and Sinopharm’s Vero Cell (BBIBP-CorV) were approved by authorities and implemented from 27 January and 27 April 2021, respectively. In the country’s National Deployment and Vaccination Plan for COVID-19, vaccination is prioritized for frontline workers, including health and sanitation workers, government workers, police, and media workers, as well as prisoners and older adults living in care homes.

Successful vaccination campaigns require authoritative vaccine-specific information and effective vaccination scheduling to reduce the use of improper vaccination methods, vaccine errors, the complexity of missed vaccination opportunities, and invalid immunisations [8]. However, two significant problems with COVID-19 vaccination campaigns have been slow uptake [9], and the dissemination of misinformation about the vaccines and vaccination in general via social media [10]. Such misinformation derives partly from the fact that vaccines work by triggering the immune system and are thus generally liable to cause adverse events (AE) when administered, with variation according to sex, age group, and presence of chronic diseases [11–14]. Some misinformation has mixed presence of such AEs with concocted effects, thereby fuelling rumour-mongering about vaccination. Beyond that, the rapid development of COVID-19 vaccines has only heightened scepticism about their readiness for use [15]. Major adverse events of the COVID-19 vaccine include pain, redness or swelling at the site of vaccine shot, fatigue, headache, muscle pain, nausea, cough, vomiting, itching, chills, myalgia, sore throat, joint pain, and can also rarely cause anaphylactic shock [16–18]. This study, estimates the incidence of adverse events, their associated factors, and attitudes about vaccine safety among frontline workers and Covishield vaccinated high-risk population. In doing so, we aimed to counter exaggerated rumours associated with COVID-19 vaccines and thereby encourage vaccination in the general public.

## Methods

### Study design

The study was designed as a two-stage, cross-section survey of randomly sampled Covishield-vaccinated individuals in eastern Nepal who were vaccinated for the first dose between 27 January and 5 March 2020.

### Setting

The study was conducted in the Dhankuta district and its periphery, specifically in a secondary hospital, two primary health centres and 36 health posts. As of 7 July 2021, only 2.7% of Nepal’s population has been fully vaccinated, largely owing to poor user confidence and limited access to vaccines.

### Participants

Our study population encompassed healthcare workers and support staff, female community health volunteers, government workers, people’s representatives, media workers, security personnel, sanitation workers, older adults living in care homes, and prisoners who received their first dose of Covishield during the first phase of Nepal’s national COVID-19 vaccination campaign. The complete list of vaccinated individuals in Dhankuta and its periphery available from Dhankuta District Health Office was used as the sampling frame, from which participants were randomly selected via lottery. The sample size was calculated with a pre-set formula [19], (Z = 2.57, p = 0.5, d = 0.05) with margins of 10% for both non-response in the first round and attrition in the second. Of 637 individuals approached, 602 completed the first round (non-response rate = 5.6%), while 516 of 602 completed the second (attrition rate = 14.3%).

### Patient and public involvement

There was no patient or public involvement in this research.

### Study tools

Data was collected using a semi-structured self-prepared questionnaire administered in two rounds following two vaccine doses from March 15 to April 15, 2021. The questionnaire was based on multiple previous studies [20–22], and was thoroughly pre-tested in a representative population of the study area. It was revised following the feedback from interviewing doctors and reviewed by the ethical review board. The questionnaire consisted of three broad sections respectively addressing participants’ socio-demographic and professional characteristics (i.e. age, gender, marital status, occupation profile, and level of education), prior disease burden and risky behaviours (i.e. existing long-term comorbidities, current medication, tobacco use, and alcohol consumption), and COVID-19 exposure and vaccination (i.e. prior COVID-19 infection of self and family, involvement in COVID-19 treatment, source of information about vaccination, initial opinions about vaccination, confidence in the vaccines, willingness to be vaccinated, vaccination coercion, vaccination date, post-vaccination symptoms, time of onset and duration of any symptoms, medications, and likelihood of receiving the second dose). After a minimum of 21 days from each dose of Covishield, we interviewed participants face-to-face with their oral and written consent. Telephone interviews were performed in the second round whenever physical access was impossible.

### Study variables

Outcome variables were the incidence, onset, duration, and severity of AEs (i.e. pain in injected arm, swelling in injected arm, fever, headache, shivering, muscle pain, joint pain, throat pain, bodily weakness, coughing, shortness of breath, common cold, sleep disturbance, loose stool, and loss of smell). Internal consistency of adverse event variables was found to be excellent (Cronbach’s $\alpha =0.9$). We obtained maximum temperature recorded for fever, frequency for loose stool, and presence or absence for loss of smell. By severity, the AEs were classified into three categories: mild (i.e. without impact on working ability), moderate (i.e. with impact on working ability without consultation or hospitalization), and severe (i.e. resulting in medical consultation or hospitalization). We also collected information on comorbidities, if any, and drugs currently being taken. Vaccine recommendation is willingness to recommend vaccination to others by the vaccinated individual following the first dose. As interview happened, at minimal, after 21 days, vaccine recommendation catches potential opinion change on vaccination due to the AEFI.

### Bias

Although vaccination was offered to the high-risk group, the decision to be vaccinated was left to individuals. That dynamic engendered self-selection, whereby people who favoured vaccination decided to be vaccinated and thus are sampled in our survey.

### Statistical analysis

Herein, we report the frequency, percentage, and confidence interval (95% CI) for sociodemographic characteristics, comorbidities, drug use, perceptions of COVID-19, and adverse events following immunisation (AEFIs), as well as the incidence, onset, duration, severity, and medication used. Quantitative data obtained from the field were coded and parsed using MS Excel and analysed in R 4.0.1. [23]. The chi-square test was used to determine associations when appropriate.

### Ethical considerations

Ethical approval was obtained from the Nepal Health Research Council (Ethical Review Board Protocol Registration No. 325/2021 P) after the submission of a brief research proposal, which was subjected to double-blind review and certified to fulfil the required protocol. All methods were performed in accordance with the relevant guidelines and regulations of NHRC. Informed consent was obtained from all the participants.

## Results

### Socio-demographic characteristics

By age, participants were from 18 to 85 years old, with a mean of 37.8 ± 13.2 years. Among the respondents, 5.9% and 21.3% reported using tobacco products and consuming alcohol, respectively (Table 1). Of the 91 participants (15.1%) with at least one comorbid illness, 39 (42.9%) had hypertension only, 14 (15.4%) had diabetes only, and 19 (20.9%) had two or more comorbid conditions (Additional file 1: Table S1). Regarding their history of COVID-19 infection, 28 participants (4.6%) reported testing positive before receiving the vaccine, while 32 (5.3%) had a history of COVID-19 infections in their family before being vaccinated (Table 1).

**Table 1:** Socio-demographic characteristics and relationship of comorbidities with AEFIs after the first dose

Variables	Category	Frequency/percent of participant	Frequency/percent^a^ of AEFI with 95% CI	p-value^b^
Total participants		602	481[79.9%, (76.7–83.1)]	
Gender	Female	183 (30.4%)	157 [85.8%, (80.7–90.8)]	0.023
	Male	419 (69.6%)	324 [77.3%, (73.3–81.3)]	
Age	18–30	222 (36.9%)	192 [86.5%, (82.0–91.0)]	0.001
	31–45	239 (39.7%)	192 [80.3%, (75.3–85.4)]	
	46–60	98 (16.3%)	67 [68.4%, (59.2–77.6)]	
	≥ 61	43 (7.1%)	30 [69.8%, (56.0–83.5)]	
Marital status	Unmarried	148 (24.6%)	129 [87.2%, (81.8–92.5)]	0.016
	Married	454 (75.4%)	352 [77.5%, (73.7–81.4)]	
Education status	Illiterate	27 (4.5%)	20 [74.1%, (57.5–90.6)]	0.412
	Primary	76 (12.6%)	57 [75%, (65.3–84.7)]	
	Secondary	205 (34.0%)	162 [79.0%, (73.4–84.6)]	
	University	294 (48.8%)	242 [82.3%, (77.9–86.7)]	
Type of occupation	Health worker	140 (23.3%)	122 [87.1%, (81.6–92.7)]	0.001
	Government worker	267 (44.3%)	205 [76.8%, (71.7–81.8)]	
	Media worker	23 (3.8%)	12 [52.2%, (31.8–72.6)]	
	Others	172 (28.6%)	142 [82.6%, (76.9–88.2)]	
Smoking	Yes	36 (5.9%)	25 [69.4%, (54.4–84.5)]	0.162
	No	566 (94.1%)	456[80.6%, (77.3–83.8)]	
Drinking alcohol	Yes	128 (21.3%)	94[73.4%, (65.8–81.1)]	0.053
	No	474 (78.7%)	387 [81.6%, (78.2–85.1)]	
Comorbid illness	Yes	91 (15.1%)	66 [72.5%, (63.4–81.7)]	0.078
	No	511 (84.9%)	415[81.2%, (77.8–84.6)]	
Prior COVID-19 infection	Yes	28 (4.6%)	22 [78.6%, (63.4–93.8)]	0.999
	No	574 (95.4%)	459[80.0%, (76.7–83.2)]	
Prior COVID-19 infection in a family	Yes	32 (5.3%)	28[87.5%, (76.0–98.9)]	0.381
	No	570 (94.7%)	453[79.5%, (76.2–82.8)]	
Involvement in treatment of COVID-19 patient	Yes	98 (16.3%)	81 [82.6%, (75.1–90.1)]	0.500
	No	504 (83.7%)	400 [79.4%, (75.9–82.9)]	

^a^Percentage was calculated for the frequency of the category variable

^b^The chi-square test was used for AEFIs observed with the first dose in each group in the study population

### Incidence of AEs following the first dose

**Fig. 1:**
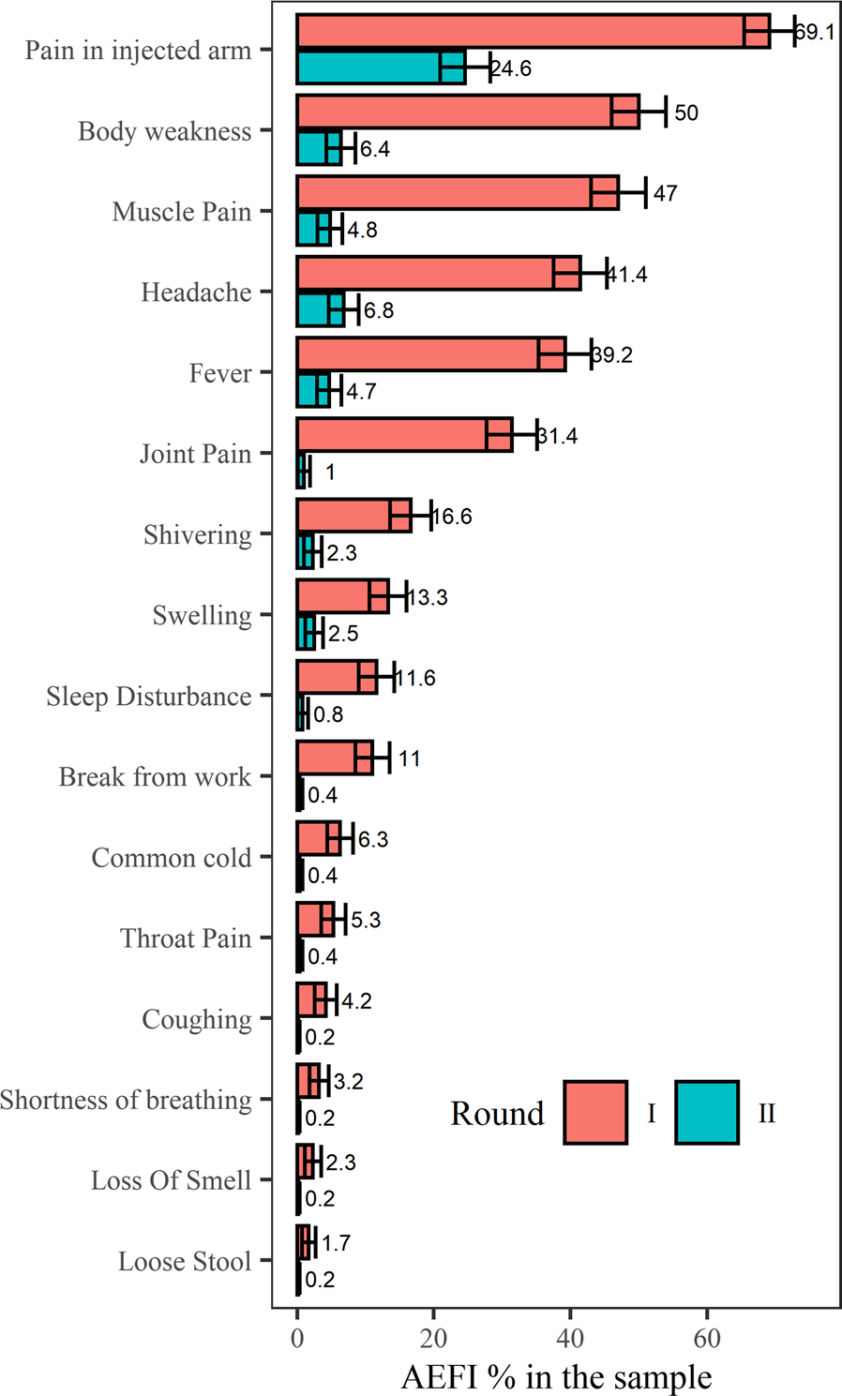
AEs following the first and second doses of Covishield (percentage = number of individual symptoms with the total number of participants in each round of vaccination). All AEFI symptoms were reported less often following the second dose. In the truncated data representing the 516 participants who participated in both rounds, fewer AEFIs were reported during the second round (χ^2^ = 13.4, *df* = 1, *p* < 0.001)

**Fig. 2:**
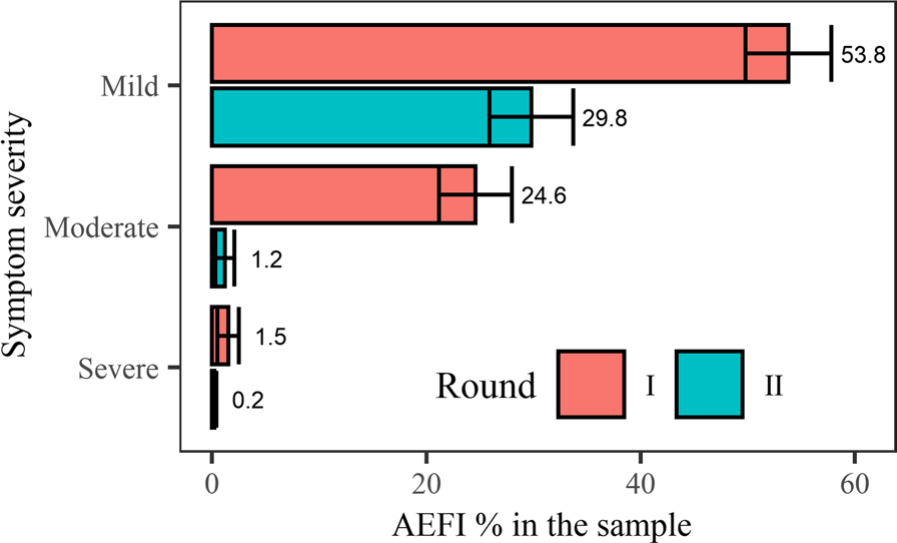
Severity of symptoms after the first and second doses of Covishield (percentage = number of individual symptoms per total respondents in each round of vaccination). AEFIs after the second dose tended to be milder than after the first dose. In the truncated data representing the 516 participants who participated in both rounds, reported AEFIs were less severe during the second round (χ^2^ = 102.7, *df* = 12, *p* < 0.001) vis-à-vis first round

After the first dose, of 602 participants, 481 (79.9%, 95% CI [76.7–83.1]) reported experiencing AEFIs, of which pain at the injection site (86.5%), bodily weakness (62.6%), muscle pain (58.8%), headache (51.8%), and fever (49.0%) were the most common complaints (Fig. 1, Additional file 1: Table S2). Two-third of complaints (67.4%, 95% CI 63.2–71.6) were for mild symptoms, and nine participants (1.9%, 95% CI 0.7–3.1) reported severe symptoms (Figs. 1, 2). The onset of AEFIs was within 12 h of vaccination for 72.8% of participants (95% CI 68.8–76.7), and they lasted more than 12 h in 76.3% (95% CI 72.5–80.1) (Additional file 1: Table S3). Of the participants who experienced AEs, 66 (13.7%, 95% CI 10.6–16.8) had to leave work, for an average break of 1.6 days (Additional file 1: Table S2).

Age was significantly associated with the incidence of AEs following the first dose; a higher incidence was found among 18–30-year-olds (χ^2^ = 16.9, *df* = 3, *p* < 0.001). In addition, AEFI severity after the first dose decreased with age (Additional file 1: Table S4). Among all the female participants, 85.8% had AEs that which is a higher (χ^2^ = 5.2, *df* = 1, p < 0.05) vis-à-vis males (77.3%). The pre-existence of comorbid illness (i.e. diabetes mellitus, hypertension, heart disease, epilepsy, kidney disease, thyroid disease, bronchitis, and bronchial asthma) was not significantly associated with the incidence of AEFIs, whereas marital status was (χ^2^ = 5.9, *df* = 1, *p* < 0.05), with unmarried individuals having a higher incidence. This effect is likely due to the age, as younger age participants are more likely to be unmarried (correlation = 0.522, t = 14.9, df = 600, p-value < 0.0001). This outcome was further supported by multivariable ordinal regression with AEFI severity as the outcome. Marriage was not statistically significant, whereas, age was a stronger predictor of AEFI severity (Additional file 1: Table S4). The incidence of AEFIs among participants who reported consuming alcohol (73.4%) was lower than among ones who did not (81.6%), and the association was statistically significant (χ^2^ = 3.7, *df* = 1, *p* < 0.05) (Table 1).

### Incidence of AEs following the second dose

Absence (n = 86) of first-round participants in the second round of the survey was not associated with vaccine opinion (χ^2^ = 0.7, *df* = 1, *p* = 0.40) and symptom severity (χ^2^ = 0.3, *df* = 2, *p* = 0.88) recorded in the first round. After the second dose, 161 of 516 participants (31.2%, 95% CI 27.2–35.2) reported experiencing AEs, 95.7% of which were mild (95% CI 92.5–98.8). The majority experienced pain at the injection site (78.9%), followed by headache (21.7%) (Additional file 1: Table S5). For 92.5% of participants (95% CI 88.5–96.6), the onset of AEFIs occurred in less than 12 h, and for 69.6% (95% CI 62.5–76.7), they lasted more than 12 h (Additional file 1: Table S6). The AEs were not significantly associated with gender, age, marital status, level of education, pre-existence of comorbid conditions, alcohol consumption, or tobacco use but were significantly associated with AEFIs with the first dose (χ^2^ = 14.3, *df* = 2, *p* < 0.001). Only two participants (1.2%) had to leave work as a result of AEFIs (Additional file 1: Table S5).

### Medication following AEFIs

In the first round, of the 125 participants (26.0%) who used medication after experiencing AEFIs, the majority (77.6%, 95% CI 70.3–84.9) used paracetamol only. Overall, the use of medication after the first dose was greater among participants with severe AEs (88.9%) than those with moderate AEs (45.9%) and mild AEs (15.1%). Following the second dose, only 12 participants (7.45%) took medication for AEs and most (91.7%, 95% CI 83.3–100) took paracetamol only (Table 2).

**Table 2:** Use of medication for AEFIs by severity

Round	Name of medication	Adverse events after COVID-19 vaccination				Percentage (%)	95% CI
		Mild	Moderate	Severe	Frequency^a^		
I	Paracetamol only	44	47	6	97	77.6	70.3–84.9
	Combination of Paracetamol and other NSAIDs^b^	5	15	2	22	17.6	10.9–24.3
	Combination of Paracetamol and drugs other than NSAIDs^c^	0	6	0	6	4.8	1.1–8.5
II	Paracetamol only	10	1	0	11	91.7	83.3–100
	Combination of Paracetamol and other NSAIDs^b^	1	0	0	1	8.3	0–16.7

^a^Total frequency of medicine use on the first round was 125 and on the second round was 12

^b^Combination of Paracetamol and NSAIDS = Flexion (Paracetamol + Ibuprofen), Flexion (Paracetamol + Ibuprofen) + Nimesulide, Nimesulide + Paracetamol

^c^Combination of Paracetamol and drug other than NSAIDS = Paracetamol + Sinex (Paracetamol + phenylpropanolamine + chlorpheniramine + caffeine), Codopar (Paracetamol + codeine phosphate), Paracetamol + Cough syrup, Paracetamol + Metronidazole, Paracetamol + Tizanidine

### Perceptions of the vaccine

The principal source of information about the vaccine was media (54.1%, 95% CI 50.2–58.1), followed by healthcare personnel (36.2%, 95% CI 32.4–40.0). Confidence in the vaccine was high, with 86.0% of participants (95% CI 83.3–88.8%) agreeing that it was safe and efficacious, whereas 10.6% (95% CI 8.2–13.0) believed that further research was needed before they could fully trust the vaccine. Such high trust was also reflected by the low number of participants (4.0%, 95% CI 2.4–5.5) who did not want to recommend vaccination to others; two-thirds (75%, 95% CI 57.7–92.3) due to doubt about the vaccine’s safety and efficacy and rest due to concerns about AEs. Vaccine recommendation following the first dose was significantly related with AEFI symptom severity in negative direction, whereas, positive initial perception of vaccine was associated with higher recommendation for vaccination (Table 3). Compared to participants whose initial opinions were that of vaccine requiring further research, participants holding opinion that vaccine were safe were 28.7 times more likely to recommend vaccine following the first dose. Most participants (96.8%, 95% CI 95.4–98.2) were interested in receiving the second dose after receiving the first. For the few participants (6.6%, 95% CI 4.6–8.6) who were coerced into vaccination, common sources of coercion were peer pressure (40%, 95% CI 24.8–55.2) and workplace regulations (37.5%, 95% CI 22.5–52.5) (Additional file 1: Table S7).

**Table 3:** Logistic regression of vaccine recommendation following the first dose with AEFI symptom severity and initial vaccine opinion

		Vaccine recommendation following the first dose			
		(1)	(2)	(3)	(4)
AEFI severity (no symptom as base category)	Mild	1.509 (0.857, 0.469)		1.291 (0.751, 0.661)	1.857 (1.208, 0.342)
	Moderate	1.020 (0.631, 0.974)		0.776 (0.503, 0.696)	0.884 (0.648, 0.866)
	Severe	0.054*** (0.044, < 0.001)		0.040*** (0.035, < 0.001)	0.062** (0.070, 0.014)
Initial vaccine opinion (need further research as base category)	Safe and effective		23.677*** (13.066, < 0.001)		28.658*** (16.961, < 0.0001)
	Unsafe and ineffective		0.429 (0.243, 0.136)		0.622 (0.427, 0.490)
*Control variables*					
Age (years)				0.989 (0.020, 0.578)	0.998 (0.021, 0.930)
Gender	Male			0.550 (0.323, 0.309)	0.486 (0.325, 0.281)
Education status (illiterate as base category)	Primary			0.962 (1.158, 0.974)	0.701 (1.138, 0.827)
	Secondary			1.250 (1.535, 0.856)	1.339 (2.177, 0.857)
	University			1.945 (2.422, 0.593)	1.975 (3.324, 0.686)
Occupation (government worker as base category)	Health worker			1.074 (0.662, 0.908)	1.208 (0.813, 0.779)
	Others			1.648 (0.946, 0.384)	2.362 (1.544, 0.189)
Constant		23.200*** (10.597, < 0.001)	4.333*** (1.388, < 0.001)	38.221** (70.371, 0.048)	3.319 (7.344, 0.588)
Observations		602	602	602	602
Log likelihood		− 93.245	− 72.012	− 91.501	− 65.245
Akaike Inf. Crit.		194.489	150.02	205.002	156.489

Standard errors and p-value in the parenthesis

***< 0.01; **< 0.05

## Discussion

### Principal findings

Widely reported after the first dose but less so after the second, AEFIs were mostly mild, with common complaints being pain at the injection site, bodily weakness, muscle pain, headache, and fever. Post-AEFIs medications were common analgesics and antipyretics sold over the counter. The AEFIs reported in the first round were found to be associated only with gender, age group, marital status, and alcohol consumption, not with comorbidity or earlier COVID-19 infection. In the second round, by contrast, AEFIs were strongly correlated with AEFIs experienced during the first round but not with other variables. Both the onset and duration of symptoms were briefer after the second dose.

Despite the public’s exposure to misinformation and rumours regarding the safety and efficacy of the vaccine, more than four-fifths of participants believed that the vaccine was safe and efficacious before they received their first dose. Nearly all participants showed interest in receiving the second dose and reported that they would recommend vaccination with Covishield to others. Vaccine recommendation was significantly associated with pre-vaccination perception about vaccine and negatively associated with AEFI severity following the first dose. The higher rate of positive response and interest among participants might relate to their very involvement in the study as individuals who were ready and willing to be vaccinated. The results might have differed had we sampled the general population.

The incidences of AEFIs were 79.9% and 31.2% after the first and second doses of Covishield, respectively, compared with 85.0% after the first dose in an earlier study [22]. Most of our participants experienced mild symptoms that resolved within a few days, which corroborates the results of an interim analysis of pooled data [24]. Although vaccines often cause AEs, the vast majority of them occur because the vaccine stimulates the body’s defenses and are not allergic in aetiology [25].

In our study, AEs were more common among people less than 50 years old. In fact, the younger the participant, the more severe their AEs tended to be. The Oxford COVID Vaccine Trial Group has reported a similar trend, which seems to be related to an exaggerated immune response in younger individuals, despite similar immunogenicity across all age groups after the second dose [26]. Although AEs were also more pronounced in women, that observation is not unusual for vaccines for influenza [27], and mirrors data about COVID-19 vaccines in the literature [28, 29]. More AEFIs have been reported in women in other studies as well and appear to be related to their greater immune response triggered by oestrogen [27]. Regarding marital status, unmarried participants reported more AEFIs, possibly because unmarried participants were 18–30 years old.

Last, the association of comorbid illness with AEFIs was not significant, which reflects what a study conducted on healthcare workers in the state of Kerala in India also revealed [30]. The lower rate of medication use after the second dose is due to the lower number of participants with moderate and severe AEFIs. Plus, paracetamol can be taken before vaccination to nullify most AEFIs [31].

### Strengths and limitations of the study

An adequate sample size, a higher response rate in the first round, a low attrition rate, a lack of missing data, and face-to-face interviews were our study’s strengths. The attrition was neither related with AEFI severity following the first dose nor with negative vaccine perception. Our sample had a higher frequency of men, which reflects the higher male employment rates in Nepal, where for every 100 employed men, there are only 59 employed women [32]. The age composition of the sample was also tilted towards the younger generation, again due to their higher frequency in the employed population. Alcohol consumption (21%) and tobacco use (5.98%) in our study population were lower than in the general population in the same age groups [33], possibly due to participants’ hesitancy to admit habits of substance use during face-to-face interviews.

Among the study’s limitations, data were collected from frontline workers and other high-risk populations in eastern Nepal. However, the study might have been more reliable had we studied AEFIs in Nepal’s general population. In addition, vaccine efficacy was not examined, not only due to the difficulty of distinguishing between symptoms developed due to immune response and allergic reaction but also because antibodies after vaccination were not estimated.

### Significance of the study

Our study’s results are valuable for designing sensitisation programs to reassure the general population about COVID-19 vaccination.

### Unanswered questions and future research

A cohort-based follow-up study is needed to better document the occurrence of long-term AEs.

## Conclusion

Despite the prevalence of AEFIs, vaccination against COVID-19 remains a vital strategy for combatting the current pandemic. In Nepal, recent data collected about AEFIs due to Covishield and their management are reassuring, and no significant association emerged between the incidence of symptoms with comorbid illness and previous COVID-19 infection. However, providing information about AEFIs and monitoring symptoms before and after vaccination should be done in the younger age group. Most AEFIs with both doses of Covishield were mild and self-limiting, and no serious AEFIs were reported at all. When medication was used, paracetamol or its combination with NSAIDs were preferred. Participants with negative prior perception regarding vaccine and those with severe AEFI following the first dose were less likely to suggest vaccination to others. Those findings can help to dispel rumours regarding the safety of Covishield and vaccines in general, as well as encourage people to be vaccinated and thereby break the chain of viral transmission.

## Supplementary Information

**Additional file 1: Table S1.** Comorbid illness of the study population. **Table S2.** Self-reported AEFIs after first dose. **Table S3.** Self-reported AEFIs onset and duration after first dose. **Table S4.** Ordered logistic regression (odds-ratio) output of symptom severity after first dose with age, gender, smoking, and presence of chronic disease. **Table S5.** Self-reported AEFIs after second dose. **Table S6.** Self-reported AEFIs onset and duration after second dose. **Table S7.** Perceptions among people after vaccination against COVID-19 after first dose.

## Data Availability

Anonymized data is available on public repository: https://github.com/TikaRamPoudel/AEFIs-Covishield.

## Abbreviations

AEs: Adverse events
AEFIs: Adverse events following immunization
CI: Confidence interval
COVID-19: Coronavirus disease of 2019
NSAIDs: Nonsteroidal anti-inflammatory drugs
