# Supplementary material for: Determinants of associated events following AZD1222 (Covishield) vaccination in a high-risk population in Nepal
Source: BMC Infect Dis. 2022 May 3;22:422. doi: 10.1186/s12879-022-07406-2 (PMC9062853; doi:10.1186/s12879-022-07406-2)
Supplement: Supplementary file 1 — Additional file 1: Table S1. Comorbid illness of the study population. Table S2. Self-reported AEFIs after first dose. Table S3. Self-reported AEFIs onset and duration after first dose. Table S4. Ordered logistic regression (odds-ratio) output of symptom severity after first dose with age, gender, smoking, and presence of chronic disease. Table S5. Self-reported AEFIs after second dose. Table S6. Self-reported AEFIs onset and duration after second dose. Table S7. Perceptions among people after vaccination against COVID-19 after first dose. [file 12879_2022_7406_MOESM1_ESM.docx]

S1 Table: Comorbid illness of the study population.

| Name of chronic disease | Frequency (N-91) | Percentage (%) | 95% CI |
| --- | --- | --- | --- |
| Hypertension | 39 | 42.9 | 32.7 - 53.0 |
| Diabetes | 14 | 15.4 | 8.0 - 22.8 |
| Multiple chronic disease ^f^ | 19 | 20.9 | 12.5 - 29.2 |
| Epilepsy | 4 | 4.4 | 0.2 - 8.6 |
| Respiratory Problem ^g^ | 4 | 4.4 | 0.2 - 8.6 |
| Thyroid Problem | 5 | 5.5 | 0.8 - 10.2 |
| Other chronic disease ^h^ | 6 | 6.6 | 1.5 - 11.7 |
| ^f^ Multiple chronic disease= More than one chronic disease reported in single participant  ^g^ Respiratory Problem= Asthma+ Bronchitis  ^h^ Other chronic disease= Kidney disease, Carcinoma Breast, Heart disease, Back Pain, Osteoporosis | | | |

S2 Table: Self-reported AEFIs after first Dose

| Symptoms | Frequency of AEFIs (N-481) | | | | | Percentage | 95% CI |
| --- | --- | --- | --- | --- | --- | --- | --- |
|  | Mild | Moderate | Severe | | Total |  |  |
| Stiffness of injection arm | | | | | | | |
| Pain | 304 | 107 | 5 | | 416 | 86.5 | 83.4 - 89.5 |
| Swelling | 66 | 13 | 1 | | 80 | 16.6 | 13.3 - 20.0 |
| Systemic symptoms | | | | | | | |
| Headache | 173 | 66 | 10 | | 249 | 51.8 | 47.3 - 56.2 |
| Shivering | 59 | 33 | 8 | | 100 | 20.8 | 17.2 - 24.4 |
| Muscle Pain | 186 | 91 | 6 | | 283 | 58.8 | 54.4 - 63.2 |
| Body weakness | 200 | 95 | 6 | | 301 | 62.6 | 58.3 - 66.9 |
| Joint Pain | 119 | 67 | 3 | | 189 | 39.3 | 34.9 - 43.6 |
| Coughing | 19 | 4 | 2 | | 25 | 5.2 | 3.22 - 7.2 |
| Shortness of breath | 12 | 5 | 2 | | 19 | 3.9 | 2.2 - 5.7 |
| Common cold | 31 | 6 | 1 | | 38 | 7.9 | 5.5 - 10.3 |
| Throat Pain | 22 | 8 | 2 | | 32 | 6.6 | 4.4 - 8.9 |
| Sleep Disturbance | 39 | 29 | 2 | | 70 | 14.5 | 11.4 - 17.7 |
| Loose Stool | 10 | | | | | 2.0 | 0.8 - 3.4 |
| Fever ^i^ | Low | Moderate | High | Unmeasured | 236 | 49.0 | 44.6 - 53.5 |
|  | 43 | 46 | 4 | 143 |  |  |  |
| Loss Of Smell ^j^ | 14 | | | | | 2.9 | 1.4 - 4.41 |
| Break from work | 66 | | | | | 13.72 | 10.65 - 16.79 |
| ^i^ Fever is categorize as low grade (99F-100F), Moderate grade (100F-103F), And High grade (>103F).  ^j^ Loss of smell is categorize as Present or absent. | | | | | | | |

S3 Table: Self-reported AEFIs Onset and Duration after first Dose

| Table 2A: Onset of AEFIs (N-481) | | | | |
| --- | --- | --- | --- | --- |
| Duration | 0-6 Hours | 6-12 Hours | 12-24 Hours | More than 24 Hours |
| frequency | 145 | 205 | 87 | 44 |
| Percent (%) | 30.1 | 42.6 | 18.0 | 9.1 |
| 95% CI | 26.0 - 34.2 | 38.2 - 47.0 | 14.6 - 21.5 | 6.6 - 11.7 |
| Table 2B: Duration of AEFIs (N-481) | | | | |
| Duration | 0-6 Hours | 6-12 Hours | 12-24 Hours | More than 24 Hours |
| frequency | 6 | 108 | 187 | 180 |
| Percent (%) | 1.2 | 22.4 | 38.9 | 37.4 |
| 95% CI | 0.26 - 2.24 | 18.72 - 26.2 | 34.5 - 43.2 | 33.1 - 41.7 |

S4 Table: Ordered logistic regression (odds-ratio) output of symptom severity after first dose with age, gender, smoking, drinking and presence of chronic disease

|  | | AEFI severity following first dose | |
| --- | --- | --- | --- |
|  |  | (1) | (2) |
| Cut 1 | | -2.883  (0.300) | -4.011  (0.656) |
| Cut 2 | | -0.360  (0.270) | -1.446  (0.635) |
| Cut 3 | | 2.824  (0.415) | 1.766  (0.707) |
| Age (years) | | 0.969***  (0.007, <0.001) | 0.965***  (0.009, <0.001) |
| Gender (Male = 1) | | 0.636***  (0.111, 0.009) | 0.675**  (0.120, 0.027) |
| Smoking | | 0.168  (0.428, 0.673) | 1.117  (0.414, 0.766) |
| Drinking | | 0.912  (0.187, 0.654) | 0.871  (0.182, 0.507) |
| Presence of chronic disease | | 1.470  (0.365, 0.120) | 1.515*  (0.381, 0.098) |
| Married | |  | 0.885  (0.196, 0.582) |
| Education (Illiterate as the base category) | Primary |  | 0.273**  (0.138, 0.010) |
|  | Secondary |  | 0.329**  (0.163, 0.025) |
|  | University |  | 0.435*  (0.215, 0.092) |
| Prior Covid-19 infection | |  | 1.476  (0.586, 0.326) |
| Prior Covid-19 infection in family | |  | 0.876  (0.313, 0.710) |
| Initial vaccine opinion (Negative) | |  | 1.409  (0.324, 0.136) |
| Pseudo R square | | 0.0240 | 0.0348 |
| Observations | | 602 | 602 |
| LR chi2 | | 30.74 | 44.55 |
| Standard errors and p-value in the parenthesis. ***<0.01; **<0.05; *<0.1. | | | |

S5 Table: Self-reported AEFIs after Second Dose

| Symptoms | Frequency of AEFIs (N-161) | | | | | Percentage (%) | 95% CI |
| --- | --- | --- | --- | --- | --- | --- | --- |
|  | Mild | Moderate | | Severe | Total |  |  |
| Stiffness of injection arm | | | | | | | |
| Pain | 125 | 1 | | 1 | 127 | 78.8 | 72.6 - 85.2 |
| Swelling | 13 |  | |  | 13 | 8.0 | 3.9 -  12.3 |
| Systemic symptoms | | | | | | | |
| Headache | 32 | 3 | |  | 35 | 21.7 | 15.4 - 28.1 |
| Shivering | 10 | 2 | |  | 12 | 7.4 | 3.4 -  11.5 |
| Muscle Pain | 24 | 1 | |  | 25 | 15.5 | 9.9 -  21.1 |
| Body weakness | 32 |  | | 1 | 33 | 20.5 | 14.3 -  26.7 |
| Joint Pain | 4 | 1 | |  | 5 | 3.1 | 0.4 -  5.8 |
| Coughing | 1 |  | |  | 1 | 0.6 | 0 -  1.2 |
| Shortness of breath | 1 |  | |  | 1 | 0.6 | 0 -  1.2 |
| Common cold | 1 | 1 | |  | 2 | 1.2 | 0 -  2.5 |
| Throat Pain | 1 | 1 | |  | 2 | 1.2 | 0 -  2.5 |
| Sleep Disturbance | 3 | 1 | |  | 4 | 2.5 | 0.1 -  4.9 |
| Loose Stool | 1 | | | | 1 | 0.6 | 0 -  1.2 |
| Fever ^i^ | Low | Moderate | High | Unmeasured | 24 | 14.9 | 9.4 - 20.4 |
|  | 8 |  |  | 16 |  |  |  |
| Loss Of Smell ^j^ | 1 | | | | | 0.6 | 0 -  1.2 |
| Break from work | 2 | | | | | 1.2 | 0 -  2.5 |
| ^i^ Fever is categorize as low grade (99F-100F), Moderate grade (100F-103F), And High grade (>103F).  ^j^ Loss of smell is categorize as Present or absent. | | | | | | | |

S6 Table: Self-reported AEFIs Onset and Duration after Second Dose

| Table 5A: Onset of AEFIs (N-161) | | | | |
| --- | --- | --- | --- | --- |
| Duration | 0-6 Hours | 6-12 Hours | 12-24 Hours | More than 24 Hours |
| frequency | 85 | 64 | 10 | 2 |
| Percent (%) | 52.8 | 39.7 | 6.2 | 1.2 |
| 95% CI | 45.0 -  60.5 | 32.2 - 47.3 | 2.5 - 9.9 | 0 – 2.5 |
| Table 5B: Duration of AEFIs (N-161) | | | | |
| Duration | 0-6 Hours | 6-12 Hours | 12-24 Hours | More than 24 Hours |
| frequency | 13 | 36 | 71 | 41 |
| Percent (%) | 8.0 | 22.4 | 44.1 | 25.5 |
| 95% CI | 3.8 - 12.3 | 15.9 - 28.8 | 36.4 - 51.8 | 18.7 - 32.2 |

S7 Table: Perceptions among people after vaccination against COVID-19 after first Dose

| Perception Variables | Levels | Frequency (N-602) | Percentage (%) | 95% CI |
| --- | --- | --- | --- | --- |
| Suggestion for Vaccination | Yes | 578 | 96.0 | 94.4 - 97.6 |
|  | No | 24 | 4.0 | 2.4 - 5.5 |
| Reason of Negative Suggestion for Vaccination | Adverse effect | 6 | 25 | 7.7 - 42.3 |
|  | Doubt regarding safety and efficacy | 18 | 75 | 57.7 - 92.3 |
| Second Dose Interest | Yes | 583 | 96.8 | 95.4 - 98.2 |
|  | No | 19 | 3.2 | 1.8 - 4.6 |
| Reason of Negative Interest in Second Dose | Adverse effect | 6 | 31.6 | 10.7 - 52.5 |
|  | Doubt regarding safety and efficacy | 13 | 68.4 | 47.5 - 89.3 |
